# Defining Key Genes Regulating Morphogenesis of Apocrine Sweat Gland in Sheepskin

**DOI:** 10.3389/fgene.2018.00739

**Published:** 2019-01-30

**Authors:** Shaomei Li, Xinting Zheng, Yangfan Nie, Wenshuo Chen, Zhiwei Liu, Yingfeng Tao, Xuewen Hu, Yong Hu, Haisheng Qiao, Quanqing Qi, Quanbang Pei, Danzhuoma Cai, Mei Yu, Chunyan Mou

**Affiliations:** ^1^Key Laboratory of Agricultural Animal Genetics, Breeding and Reproduction of Ministry of Education, College of Animal Science and Technology, Huazhong Agricultural University, Wuhan, China; ^2^Qinghai Academy of Animal Science and Veterinary Medicine, Xining, China; ^3^Sanjiaocheng Sheep Breeding Farm, Haibei, China; ^4^Animal Husbandry and Veterinary Station, Haixi, China

**Keywords:** sweat gland, wool follicle, skin, morphogenesis, WNT, TGF-β, transcriptome

## Abstract

The apocrine sweat gland is a unique skin appendage in humans compared to mouse and chicken models. The absence of apocrine sweat glands in chicken and murine skin largely restrains further understanding of the complexity of human skin biology and skin diseases, like hircismus. Sheep may serve as an additional system for skin appendage investigation owing to the distributions and histological similarities between the apocrine sweat glands of sheep trunk skin and human armpit skin. To understand the molecular mechanisms underlying morphogenesis of apocrine sweat glands in sheepskin, transcriptome analyses were conducted to reveal 1631 differentially expressed genes that were mainly enriched in three functional groups (cellular component, molecular function and biological process), particularly in gland, epithelial, hair follicle and skin development. There were 7 Gene Ontology (GO) terms enriched in epithelial cell migration and morphogenesis of branching epithelium that were potentially correlated with the wool follicle peg elongation. An additional 5 GO terms were enriched in gland morphogenesis (20 genes), gland development (42 genes), salivary gland morphogenesis and development (8 genes), branching involved in salivary gland morphogenesis (6 genes) and mammary gland epithelial cell differentiation (4 genes). The enriched gland-related genes and two Kyoto Encyclopedia of Genes and Genomes pathway genes (WNT and TGF-β) were potentially involved in the induction of apocrine sweat glands. Genes named *BMPR1A, BMP7, SMAD4, TGFB3, WIF1*, and *WNT10B* were selected to validate transcript expression by qRT-PCR. Immunohistochemistry was performed to localize markers for hair follicle (SOX2), skin fibroblast (PDGFRB), stem cells (SOX9) and BMP signaling (SMAD5) in sheepskin. SOX2 and PDGFRB were absent in apocrine sweat glands. SOX9 and SMAD5 were both observed in precursor cells of apocrine sweat glands and later in gland ducts. These results combined with the upregulation of BMP signaling genes indicate that apocrine sweat glands were originated from outer root sheath of primary wool follicle and positively regulated by BMP signaling. This report established the primary network regulating early development of apocrine sweat glands in sheepskin and will facilitate the further understanding of histology and pathology of apocrine sweat glands in human and companion animal skin.

## Introduction

Human skin is the largest organ that covers the body surface and balances heat and protects against assaults from the environment. It contains different subtypes of appendages including hair follicles, nails, sebaceous glands and sweat glands that display diverse histological structures and regional localizations in different body parts. The apocrine and eccrine sweat glands are two types distributed across the human body. Though both sweat glands have similar structures consisting of ductal and secretory portions, they do have different functions and locations (Sato et al., 1989). The eccrine sweat glands are generally found on hairless body regions, especially on the palms and soles (Sato et al., 1989), with slim ducts and small secretory portions that secrete water and electrolytes directly to the surface of the human body (Lobitz and Dobson, 1961; Yanagawa et al., 1986). In contrast, the apocrine sweat glands are connected to the upper part of hair follicles in hairy regions such as axilla and perineum (Sato et al., 1989), with short and thick ducts and large secretory coils that release viscous liquid (water, electrolytes, protein, lipids, and steroids to the opening of hair follicles) (Sato et al., 1989; Wilke et al., 2007). Under disease conditions with hircismus, the secretions from apocrine sweat glands are turned from originally odorless to odorous compounds by bacterial enzymes on the skin’s surface (Shehadeh and Kligman, 1963; Preti and Leyden, 2010). A functional allele (538G > A) in the ATP-binding cassette C11 (ABCC11) gene was reported to highly associate with human earwax type (wet or dry) and axillary odor (Yoshiura et al., 2006; Toyoda et al., 2009, 2017; Martin et al., 2010). The increased expression of ABCC11 in apocrine sweat glands was detected more in the myoepithelial cells of the secretory portions in individuals with GG genotype than those of AA genotype (Toyoda et al., 2017). Though these reports suggested an interesting correlation between the ABCC11 gene and the axillary odor caused by apocrine sweat glands in human skin, the actual mechanisms underlying it remain unknown. More information related to apocrine sweat glands may assist the diagnosis and even practical treatment of this skin disease.

The apocrine sweat gland marks a big difference in the skin between humans and animal models (chicken and mouse) in that murine and chicken skin are exclusively lacking in apocrine sweat glands. Additionally, its absence in murine and chicken skin restrains its related investigations. Sheepskin may represent an additional system to gain basic information about apocrine sweat glands. The histological structure of apocrine sweat glands in the armpit skin are similar to those of sheep body skin (Sato et al., 1989; Rogers, 2006). Hence, the general knowledge of sheepskin would facilitate further understanding of human apocrine sweat glands under normal and diseased conditions.

Previous studies regarding sweat glands mainly focused on the eccrine sweat glands by detecting *KRT* gene expression in human embryos (Hashimoto et al., 1965; Sun et al., 1979; Moll and Moll, 1992) and elucidating the molecular mechanisms of morphogenesis and development in mouse models (Kunisada et al., 2009; Cui et al., 2014; Lu et al., 2016). Several signaling pathways including wingless-related integration site (WNT), ectodysplasin A receptor (EDAR), bone morphogenetic proteins (BMP), sonic hedgehog (SHH), were shown to regulate the initiation and maturation of eccrine sweat glands (Kunisada et al., 2009; Cui et al., 2014; Lu et al., 2016).

In *Eda*-null (tabby) mice, no eccrine sweat glands were formed throughout the embryonic stage in mouse paw skin (Kunisada et al., 2009). *β-catenin* conditional knockout mice showed complete blockage of eccrine sweat gland formation from E15.5 to birth before the unexpected death of the mice (Cui et al., 2014). *Wnt10a* mutant mice developed normal prenatal eccrine sweat gland germs but failed to form sweat ducts postnatally (Xu et al., 2017). Hence, *Wnt10a*/*β-catenin* mainly regulates the maturation of eccrine sweat glands in postnatal life. The BMP pathway has been reported to play a positive role in determining the glandular fate during the induction stage of eccrine sweat gland. In *Bmpr1a* conditional knockout mice, the eccrine sweat glands were converted to hair follicle-like structures (Lu et al., 2016) and the density of eccrine sweat glands was reduced in *Bmp5* null mouse skin (Lu et al., 2016). The cross-talk of BMP and SHH spatiotemporally determined the subtypes of skin appendages, either hair follicles or eccrine sweat glands. A high BMP signal in mesenchyme and a low SHH signal in the epidermis engaged the glandular fate decision just before the initiation of eccrine sweat gland development (Lu et al., 2016). This mechanism was also observed in other ectodermal glands (mammary and meibomian) and chicken digestive epithelia formation (Roberts et al., 1998; Narita et al., 2000; Mayer et al., 2008; Huang et al., 2009a). These findings highly suggest that inhibiting BMP signaling favors hair follicle cell fates, whereas active BMP signaling promotes glandular cell fates. In addition, the eccrine sweat gland density was also shown to be determined by the expression of homeodomain transcription factor engrailed 1 (*En1*) in murine footpad skin (Kamberov et al., 2015).

Though the eccrine sweat glands gained most of the research interest, the understanding of the apocrine sweat glands was relatively restricted to the physiological and pathological descriptions of humans or companion animals, owing to the absence of apocrine sweat glands in mouse and chicken skin (Leyden et al., 1981; Kalaher et al., 1990; Morandi et al., 2005; Baharak et al., 2012; Fujiwara-Igarashi et al., 2017). Until now, there is little in the literature related to the morphogenesis and development of apocrine sweat glands.

Previously, sheepskin attracted researchers to decipher the regulatory mechanisms of wool follicle and skin development in embryonic stages and in postnatal seasonal wool growth. The induction of primary wool follicles in coarse wool sheepskin during early embryonic stages and the morphogenesis of secondary wool follicles in merino sheepskin were investigated by exploring the interaction network of long non-coding RNAs (lncRNAs) and mRNAs, including a series of lncRNAs and WNT, BMP, EDAR, and FGF signaling pathways (Yue et al., 2016; Nie et al., 2018). The microRNA profiles identified candidates (miRNA-143, miRNA-10a, let-7i) potentially regulating the different wool follicle growth patterns with small, medium or large waves in Hu sheepskin, and a series of new microRNAs during the wool follicle seasonal growth cycling in sheepskin (Liu et al., 2013, 2014; Lv et al., 2016; Gao et al., 2017). These studies focused mainly on the morphogenesis of primary and secondary wool follicles and the regulation of wool fiber thickness. The existence of apocrine sweat glands in sheepskin is a great advantage for obtaining a deep understanding of the complexity of skin biology. Our current study aimed to explore the dynamic gene regulatory network and potential candidate genes governing the apocrine sweat gland induction in skin using sheepskin as a model system. This result will add general knowledge regarding the histological and molecular changes during the apocrine sweat gland morphogenesis and contribute to the further understanding of apocrine sweat gland development in skin of normal or diseased human or companion animals.

## Materials and Methods

### Experimental Animals

Coarse wool sheep (Tibetan carpet wool sheep) fetuses were randomly collected from a local abattoir in Qinghai Province of China as described previously (Nie et al., 2018). Briefly, the discarded fetuses were rescued and immediately placed in PBS. The dorsal skin was dissected and divided into two parts. One part was fixed in 4% paraformaldehyde at 4°C and the other part was frozen in liquid nitrogen for RNA extraction. The individuals (approximately 120 individuals) at unspecified embryonic stages were randomly collected for the determination of developmental stage by H&E (hematoxylin and eosin) staining. All experiments on animals were approved by the Standing Committee of Hubei People’s Congress and the ethics committee of Huazhong Agricultural University.

### H&E Staining

To identify the developmental stages of wool follicles and apocrine sweat glands in embryonic sheepskin, a series of fixed sheep dorsal skin samples were dehydrated with gradient alcohol, processed in paraffin and cut into 5 μm sections, according to the standard procedures. Then dorsal skin sections were processed into dewaxing and H&E (hematoxylin and eosin) staining. The stained skin sections were photographed and grouped into different developmental stages based on the structures of wool follicles and apocrine sweat glands as described in previous reports (Rogers, 2006).

### Transcriptome Sequencing and Differentially Expressed Genes Analyses

Total RNA was extracted using TRIzol reagent from six sheepskin samples and RNA integrity was assessed using the RNA Nano 6000 Assay Kit with the Bioanalyzer 2100 system (Agilent Technologies, CA, United States). The sequencing library was constructed at Novogene (Beijing, China) using a NEBNext^®^ Ultra^TM^ RNA Library Prep Kit for Illumina^®^ (NEB, United States) following the manufacturer’s procedures. The library quality was assessed on the Agilent Bioanalyzer 2100 system. After cluster generation, the libraries were sequenced on an Illumina Hiseq platform (Hiseq X ten) with 150 bp paired-end reads.

The original sequenced reads were evaluated for data quality and then clean reads were mapped to the sheep reference genome (version: Oarv3.1) by Hisat2. HTSeq v0.9.1 was used to count the reads numbers mapped to each gene. The FPKM of each gene was then calculated to estimate gene expression level (Trapnell et al., 2010). After standardizing and testing the read counts, the differentially expressed genes (DEGs) were obtained. For biological replicates, genes with an adjusted *P*-value < 0.05 and | log2 (Fold change) | > 1 were set as the threshold for differential expression (Supplementary Table S1).

### GO Term, KEGG Enrichment and PPI Analyses of Differentially Expressed Genes

Gene Ontology (GO) enrichment analyses of DEGs were implemented by the GOseq R package. GO terms with corrected *P*-values less than 0.05 were considered significantly enriched by differential expressed genes (Supplementary Table S2). KOBAS software was used to test the statistical enrichment of differential expression genes in Kyoto Encyclopedia of Genes and Genomes (KEGG) pathways (Supplementary Table S3). Protein–protein interaction analyses of DEGs were based on the commonly used STRING database, then Cytoscape software was used to realize the visualization of the interaction network (Shannon et al., 2003).

### Quantitative Real-Time PCR (qRT-PCR) Validation

Several differentially expressed mRNAs were selected and confirmed by qRT-PCR with GAPDH used as an internal reference. qRT-PCR was carried out with a Roche LightCyclerR 96 using iTaqTM Universal SYBRRGreen Supermix (Bio-Rad, United States). The amplification procedures were held at 95°C for 5 min initially, followed by 45 cycles of 95°C for 15 s and 60°C for 1 min. Quantification of mRNAs was performed using the 2^-ΔΔCt^ method with average cycle thresholds. The qRT-PCR data were generated from three independent samples per stage and statistically analyzed using Student’s *t*-test (*n* ≥ 3).

### Immunohistochemistry

Immunohistochemistry was applied to detect the expression pattern of skin appendage markers. The skin was dehydrated with ethanol, embedded in paraffin and sectioned at 5 to 6 μm thickness. The sections were dewaxed, processed to antigen retrieval and incubated with primary antibodies (Sox2, mouse, Santa Cruz,1:200; Sox9, mouse, Abcam, 1:200; pSmad5, Rabbit, Abcam,1:800; Pdgfrb, Rabbit, Abcam, 1:400) at 4°C overnight. The secondary antibody from the immunological kit (Proteintech, China) was incubated for 1 h at room temperature. Visualization was performed by using DAB staining (1:50) followed by hematoxylin counter-staining. Experiments were repeated at least twice.

## Results

### The Morphological Characterization of Developing Wool Follicles and Apocrine Sweat Glands in Coarse Wool Sheep Back Skin

**Figure 1:**
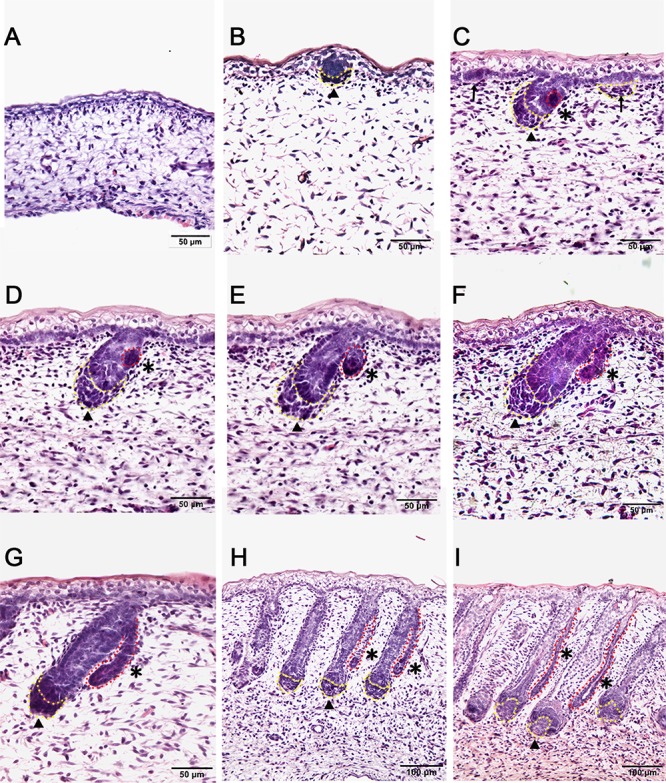
Dynamic diagram of morphogenesis and development of wool follicles and apocrine sweat glands in embryonic coarse wool sheep. **(A)** The skin is homogeneous with thin epidermal and dermal layers before the initiation of skin appendages; **(B)** The first skin appendage, the primary wool follicle, is initiated to form epidermal placodes and associated dermal condensates as marked in a yellow dashed line; **(C)** The second skin appendage, the apocrine sweat gland is inducted at the lateral side of primary wool follicle epidermal peg. At the same time, the secondary wool follicle is initiated in between the primary wool follicles. The precursor/progenitor cells of apocrine sweat glands are marked with a red dashed line; **(D)** The precursor/progenitor cells of apocrine sweat glands exhibit early signs of the branching point from the lateral side of the outer root sheath of primary wool follicles; **(E)** The germ or small ductal bud of apocrine sweat glands protrudes and forms from the upper part of primary wool follicles. The primary and secondary wool follicles continue to grow downward into the dermis; **(F–H)** The apocrine sweat gland and wool follicle gradually elongate and extend into the dermis. The apocrine sweat gland grows and extends from germ to be a slim duct-like structure at the angle parallel to the primary wool follicle peg; **(I)** The primary wool follicle becomes mature with clear dermal papilla and matrix compared to the previous stage (Figure H). The ductal portion of the apocrine sweat gland gradually extends and forms a slim tube-like structure with an emerging cavity as indicated as asterisk. **A–G** Bar, 50 μm; **H** and **I** Bar, 100 μm.^∗^, apocrine sweat gland; 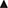, dermal condensates or dermal papilla; ↑, secondary hair follicle; dashed line in yellow, dermal papilla or dermal condensate; dashed line in red, apocrine sweat gland.

In this study, the Tibetan carpet wool sheep, a typical coarse wool sheep, was chosen for detailed investigation of the early development of apocrine sweat glands in skin. A series of sheep back skin sections were used to determine the induction and morphogenesis of apocrine sweat glands. The wool follicles and apocrine sweat glands were observed to occur sequentially based on histological H&E stain (Figure 1A–I). From the homogeneous thin skin layers (Figure 1A) to the appearance of skin appendages (Figure 1I), the obvious morphological changes were the thickening of the dermal and epidermal layers and the occurrence of wool follicle pegs, apocrine sweat glands and sebaceous glands. The first appendage that appeared in skin was the primary wool follicle that developed the placode and associated dermal condensation (Figure 1B) from the thin skin (Figure 1A), later sequentially grew downward to the dermis, elongated and progressed to maturation (Figure 1C–I). The first sign of apocrine sweat glands was observed as several cells were tightly packed and located on lateral side of the epidermal compartment of the primary wool follicle peg, indicating the occurrence of the precursor/progenitor cells of apocrine sweat glands (Figure 1C). As the cells proliferated and differentiated, these small cell patches gradually developed a small germ and later extended into the dermis to form the slim and long gland duct (Figure 1E–I). The elongation of gland ducts was directed at the angles that were closely parallel to wool follicle pegs as detected in Figure 1G–I. At this stage, the dermal condensates of primary wool follicles were encapsulated to become dermal papilla, and the sweat gland duct cavity was visible and surrounded by two layers of cells (Figure 1I). These stained sections clearly stated the initiation, budding, elongation and ductal cavity formation of apocrine sweat glands in prenatal coarse wool sheepskin.

### Differentially Expressed Genes Involved in the Elongation of Wool Follicle Peg and Induction of Apocrine Sweat Glands in Coarse Wool Sheepskin

To understand the induction of apocrine sweat glands, two particular stages that corresponded to the pre-gland (stage TF1b, Figure 1B) and gland budding stage (stage TF2a, Figure 1E) to form ductal portions (Rogers, 2006) were selected to perform RNA sequencing. The morphological changes between two selected stages were the epidermal and dermal thickening, follicle germ elongation and apocrine sweat gland budding.

**Figure 2:**
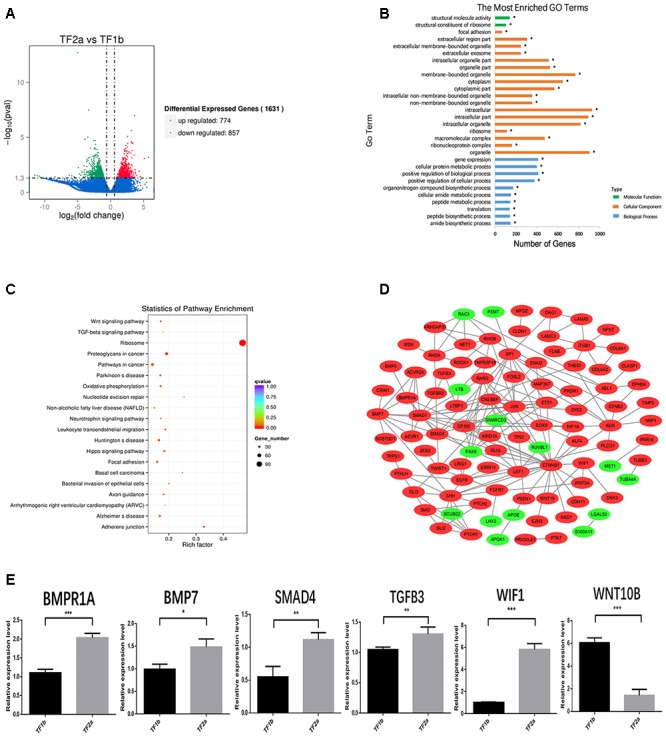
The outcome of bioinformatics analyses of RNA sequencing data of sheepskin with qRT-PCR validation. **(A)** Volcano plot displaying the mRNA transcripts enriched between stage TF2a and stage TF1b of the prenatal sheepskin. The DEGs were enriched to compare the budding stage (TF2a) with the pre-gland stage (TF1b) of apocrine sweat glands in coarse wool sheepskin (*n* = 3) as shown in the volcano plot. There were 1631 differentially expressed transcripts, including 774 upregulated (right, red) and 857 downregulated (left, green), between these two groups. The criteria set up for the enrichment are | log2 (fold change) | > 1 and *P* value (*P* < 0.05); **(B)** The top 20 GO terms are presented in the enrichment analyses of differentially expressed mRNA transcripts between apocrine sweat gland induction stages (TF2a vs. TF1b) in coarse wool sheepskin. A total of 786 terms were significantly enriched (*P* < 0.05) in the categories of biological process (blue), molecular function (green), and cellular components (yellow red). **(C)** The top 20 KEGG pathways are displayed in the enrichment analyses of differentially expressed mRNA transcripts in apocrine sweat gland stages (TF2a vs. TF1b) in coarse wool sheepskin. The top 20 out of 248 terms of differently expressed mRNA transcripts were grouped and displayed (*P* < 0.05). Of those, three signaling pathways (WNT, TGF-β, and Hippo signaling pathways), focal adhesion and adherent junctions were potentially involved in histological changes during the morphogenesis of apocrine sweat gland (TF2a vs. TF1b) in coarse wool sheepskin. **(D)** The mRNA–mRNA interaction networks were constructed by using the potential candidate genes involved in the development of skin, wool follicles and glands. The WNT, TGF-beta, and SHH signaling pathways were clearly grouped. Another two groups networked by KDR and ITGB1 were potentially involved in basement membrane and cell proliferation. **(E)** A total of 6 DEGs were selected for quantitative real-time PCR (qRT-PCR) validation. The expression patterns of *BMP1A*, *BMP7*, *SMAD4*, *TGFB3*, *WIF1*, and *WNT10B* are consistent with the tendency of mRNA sequencing results by using the 2^-ΔΔCt^ method and GAPDH as internal control. Data are presented as mean ± SD (*n* ≥ 3). ^∗^*P* < 0.05, ^∗∗^*P* < 0.01 ^∗∗∗^*P* < 0.001 (Student’s *t*-test).

The sequencing data were processed for bioinformatics analyses. The criteria set up to enrich DEGs was | log2 fold change| > 1 and *P* < 0.05. A total of 1631 genes including 774 upregulated and 857 downregulated genes exhibited significant expression changes in the stage TF2a vs. the stage TF1b target group (Figure 2A). All the DEGs were compared with the published gene lists enriched in different compartments of P5 mouse skin (Sennett et al., 2015). The overlapping genes between these two data sets were picked up and presented in Table 1. Since the mouse back skin contains no sweat glands, the listed genes shared between mouse and sheep back skin were shown to highly associate with skin and hair/wool follicle development represented by 22 genes for the epidermis, 8 genes for dermal fibroblasts, 12 genes for the outer root sheath, 3 genes for matrix, 19 genes for melanocyte, 34 genes for dermal papilla, 10 genes for transit amplifying cells and 12 genes for hair follicle stem cells (Table 1). These results suggest that the regulatory genes for prenatal wool follicles and sheepskin were partially conserved with those of postnatal murine hair follicles and skin.

**Table 1:** The overlapped differentially expressed genes (DEGs) specific for different compartments of skin are presented by comparing the dataset of sheep prenatal skin with that of murine P5 dorsal skin.

Compartments of skin	Number	Genes
Epi	22	ANXA4 CST6 DEFB1 EMC9 IL20RA KLF3 KRTDAP LGALS3 LRP4 LY6D MGST2 MST1 NET1 PARP10 S100A11 SBSN STAP2 TGFBI TSPO TST TUBA4A WNT16
ORS	12	DAPK1 FLRT2 FNDC1 GPC4 LAMA5 LGR5 LHX2 MPDZ MYH9 PLA2G7 SOX9 TIMP3
Mx	3	FABP5 TGM1 RASGEF1B
Mc	19	ARSA B2M COMMD4 DKK3 DTNBP1 ETS1 GLUL LBH LITAF NKD1 NPC2 PRDX3 RAB5B SEMA6A STX7 TUBB3 VAT1 VPS26A ZHX2
DF	8	ADAMTS15 ADAMTS18 CD34 COL6A1 COL6A2 OLFML2B S100A4 TWIST2
DP	34	ABL1 BMP3 CDH11 CYR61 EPHX2 FGFR1 GABRE GEM HIF3A IZUMO4 LAMC3 LTBP1 LYNX1 MASP1 MRC2 NDNF NOL3 OSR1 PAPPA2 PEMT PRR16 PTCH1 RSPO3 SCUBE2 SMARCD3 SNAI2 SOSTDC1 SPON1 SSC5D TRPS1 UBA7 VCAN WIF1 ZIC4
TAC	10	ATG9B EFNB2 GLI3 KDM2B LEF1 PTCH2 RASGEF1B SLC40A1 TGM1 TLE3
HF-SC	12	ADAMTS17 APOE CRIM1 FOXI3 FOXO6 GPC4 KRT13 LRIG1 PTHLH RGS2 SOX9 TGFB3

*DEGs: Differently expressed genes; HF: Hair follicle; Epi: Epidermis; ORS: Outer Root Sheath; Mc: Melanocytes; Mx: Matrix; DF: Dermal Fibroblasts; DP: Total dermal papilla cells; TAC: Transit amplifying cells; HF-SC: Bulge stem cell precursors (Sennett et al., 2015)*.

### Enriched GO Term and KEGG Pathway Analyses of the Differentially Expressed Genes

The 1631 DEGs were processed to GO term and KEGG enrichment analyses. The most enriched GO terms were biological process, cellular component and molecular function, including organelle (represented by CST6, APOA1, and MKI67), gene expression (represented by MST1) and structural molecule activity (represented by TUBA4A) (Figure 2B). Further details revealed that the GO terms were highly enriched in three categories: hair follicle and skin development, gland development and epithelial development. Two GO terms were highly associated with hair follicle development (14 genes enriched) and skin development (33 genes enriched) (Table 2). The enrichment of 5 GO terms for epithelial differentiation, migration and the branching process represented mammary gland epithelial cell differentiation (4 genes enriched), epithelial cell migration (27 genes enriched), morphogenesis of a branching epithelium (24 genes enriched), morphogenesis of an epithelial fold (8 genes enriched), morphogenesis of an epithelial bud (5 genes enriched), embryonic epithelial tube formation (23 genes enriched) and branching morphogenesis of an epithelial tube (21 genes enriched) (Table 2). Most of the enriched genes showed an upregulated expression trend. These terms highly suggest that during the two stages applied for RNA sequencing, the epithelia displayed the prominent biological functions, either for the elongation of the epidermal compartments of wool follicle pegs or the morphogenesis of the apocrine sweat gland ducts. Interestingly, a total of 5 GO terms were shown to regulate the gland morphogenesis (20 genes enriched), gland development (42 genes enriched), salivary gland morphogenesis and development (8 genes enriched), branching involved in salivary gland morphogenesis (6 genes enriched) and mammary gland epithelial cell differentiation (4 genes enriched).

**Table 2:** The GO terms are specifically enriched in skin and hair follicle development, epithelial development and gland development.

Description	Number	Genes
Salivary gland morphogenesis and development	8	BMP7 DAG1 EGFR FGFR1 LAMA5 NRP1 SNAI2 TGFB3
Branching involved in salivary gland morphogenesis	6	BMP7 DAG1 FGFR1 LAMA5 NRP1 SNAI2
mammary gland epithelial cell differentiation	4	HIF1A, SMO, PTCH1, LBH
Gland development	42	ABL1 **APOA1** ARHGAP35 ARID5B BMP7 BMPR1A CTNNB1 **COBL** DAG1 EGFR EZH2 FGFR1 GLI2 GLI3 HIF1A JUN LAMA5 LBH LEF1 **LHX3 MKI67** MSN **MST1** NRP1 **PAX8** PSEN1 PTCH1 RARG **RHBDD3** SMAD4 SMO SNAI2 SOCS2 SOSTDC1 SOX9 SP3 SULF2 **TAF10** TGFB3 TGFBR2 WLS WNT5A
Gland morphogenesis	20	BMP7 DAG1 EGFR FBR2 FGFR1 GLI2 GLI3 LAMA5 **MKI67** MSN **MST1** NRP1 PTCH1 RARG SNAI2 SOSTDC1 SOX9 SULF2 TGFB3 WNT5A
Hair follicle development	14	APCDD1 CTNNB1 EGFR LAMA5 LGR4 LGR5 LHX2 LRP4 SMAD4 SMO SOSTDC1 SOX9 TNFRSF19 WNT5A
Skin development	33	**ALOX12B** APCDD1 ARRDC3 CLDN1 CTNNB1 CYP26B1 **DACT2** EGFR ERRFI1 FLNB FOSL2 FRAS1 H2AFY2 LAMA5 LGR4 LGR5 LHX2 LRP4 **LTB** PSEN1 PTCH2 PTGES3 ROCK1 SMAD4 SMO SOSTDC1 SOX9 TNFRSF19 **TGM1** WDR48 WNT16 WNT5A ZFP36L1
Embryonic epithelial tube formation	23	ABL1 ARHGAP35 ARID1A BMP7 **COBL** CTNNB1 HIF1A KDM2B LHX2 LUZP1 OSR1 **PAX8** PRICKLE1 PTCH1 PTK7 RARG RGMA SEMA4C SOX9 TMED2 TWIST1 WNT5A ZEB2
Epithelial cell migration	27	ADGRA2 **APOA1** **APOE** ARSB EMP2 EFNB2 ETS1 EPHB4 CLASP1 FGFR1 HIF1A ITGB1 JUN KDR KLF4 LOXL2 MYH9 NRP1 PLCG1 **RAB25** RHOB **S100A2** SOX9 TGFBR2 THBS1 WNT5A ZEB2
Branching morphogenesis of an epithelial tube	21	ACVR1 BMP7 CTNNB1 DAG1 DLG5 EDNRA GLI GLI3 LAMA5 LEF1 LGR4 MMP14 NPNT NRP1 **PAX8** PTCH1 SMAD4 SMO SOX9 TGFBR2 WNT5A
Morphogenesis of a branching epithelium	24	ACVR1 BMP7 CTNNB1 DAG1 DLG5 EDNRA FGFR1 GLI2 GLI3 LAMA5 LEF1 LGR4 MMP14 NPNT NRP1 **PAX8** PTCH1 RSPO3 SMAD4 SMO SNAI2 SOX9 TGFBR2 WNT5A
Morphogenesis of a branching structure	25	ACVR1 BMP7 CTNNB1 DAG1 DLG5 EDNRA FGFR1 GLI2 GLI3 LAMA5 LEF1 LGR4 MMP14 NPNT NRP1 **PAX8** PRDM1 PTCH1 RSPO3 SMAD4 SMO SNAI2 SOX9 TGFBR2 WNT5A
Morphogenesis of an epithelial fold	8	BMP7 CTNNB1 EGFR GLI2 HIF1A LUZP1 SOSTDC1 WNT5A
Morphogenesis of an epithelial bud	5	BMP7 CTNNB1 GLI2 SOSTDC1 WNT5A

*The bold black font represents the downregulated DEGs*.

The KEGG database was used to refine the potential signaling pathways in our data. The top 20 of the 248 enriched KEGG pathways were selected and presented in Figure 2C. Of those, the WNT (19 genes enriched), TGF-β (14 genes enriched) and Hippo (25 genes enriched) signaling pathways were the most promising candidates and were highly correlated to the morphological changes between the selected two developmental stages (Table 3). In addition, the Hedgehog signaling pathway (9 genes enriched), which ranked as 21 in pathway enrichment, was another candidate regulating the morphological changes during the stages selected (Table 3). This observation is highly consistent with the enrichment of GO terms in positive regulation of cellular and biological processes (Figure 2B). Additionally, the enriched gland-related genes in 5 GO terms and 2 KEGG pathway genes were the potential candidates involved in the induction of apocrine sweat glands in coarse wool sheepskin.

**Table 3:** The DEGs enriched in three signaling pathways are potentially related to sweat gland development.

Signaling Pathway	Number	Genes
WNT	19	CREBBP CTNNB1 EP300 GPC4 JUN LEF1 MAP3K7 NKD1 PRICKLE1 PSEN1 RAC3 RHOA **RUVBL1** SMAD4 TP53 WIF1 **WNT10B** WNT16 WNT5A
TGF-beta	14	ACVR1 ACVR2A BMP7 BMPR1A CREBBP EP300 RHOA ROCK1 SMAD1 SMAD4 SP1 TGFB3 TGFBR2 THBS1
Hedgehog	9	GLI2 GLI3 PTCH1 PTCH2 SHH SMO **WNT10B** WNT16 WNT5A
Hippo	25	ACTB ACTG1 BMP7 BMPR1A CCND1 CTNNA1 CTNNB1 FRMD6 GLI2 LEF1 PPP1CB PPP1CC SMAD1 SMAD4 SNAI2 TGFB3 TGFBR2 **WNT10B** WNT16 WNT5A WWTR1 YWHAE YWHAG YWHAQ YWHAZ

*The bold black font represents the downregulated DEGs*.

### Construction of Candidate Gene Interaction Network Functioned in the Elongation of Wool Follicle Pegs and the Induction of Apocrine Sweat Glands in Coarse Wool Sheepskin

Several DEGs were used to construct an mRNA–mRNA interaction network (Figure 2D). The genes potentially regulating hair and skin development, gland development, epithelial development (Tables 1, 2) and 4 signaling pathway genes (Table 3) were all applied for network construction (Figure 2D). The gene *ITGB1* established a small network to regulate the skin dermal fibroblast (*COL6A1* and *COL6A2*) and epidermal development (*FLNB*, *LAMC3*, and *LAMA5*). The gene *KDR*, also named *VEGF*, established the network for regulating epithelial migration and branching. The TGF-β (*BMP7*, *BMPR1A*, *SMAD1*, and *SMAD4*), WNT (*CTNNB1* and *LEF1*) and SHH (*SHH* and *GLI3*) signaling pathways that indicate important regulation of gland and epithelial branching development established the complex networks in the pathway itself and as well as crosstalk among different pathways. For instance, *CTNNB1* was shown to interact with *SMAD1* and *SHH* has connection with *BMP7*, *EGFR*, *FGFR1*, and *LEF1* (Figure 2D).

### Validation of Potential Candidate Genes Functioned in Wool Follicle Peg Elongation and Apocrine Sweat Gland Induction

A total of 6 genes were selected to evaluate the RNA sequencing results by the qPCR technique. Of those, *BMP7*, *BMPR1A*, *SMAD4*, *WIF1*, and *TGFB3* showed increased expression in the apocrine sweat gland budding stage, while *WNT10B* displayed decreased expression. The expression tendency of these genes is consistent with the RNA sequencing results (Figure 2E).

**Figure 3:**
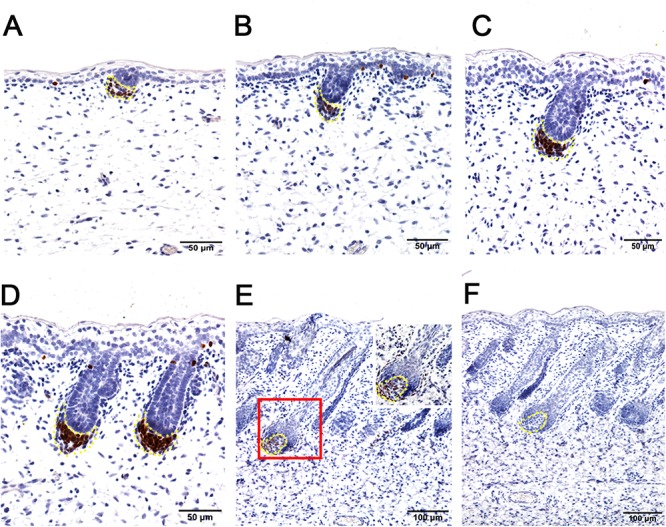
Hair follicle marker SOX2 is negative in apocrine sweat glands in prenatal sheepskin as detected by immunohistochemistry. SOX2 is specifically localized in the dermal condensates/dermal papilla of primary wool follicles, not in those of secondary wool follicles and apocrine sweat glands. **(A–E)** The strong positive signals of SOX2 antibody staining are restricted in the dermal condensates associated with the primary wool follicle placodes **(A–D)** in early stages and in the dermal papilla in later stage **(E)**. The secondary wool follicles and apocrine sweat glands are all negative for SOX2 immunohistochemistry. **(F)** The negative control is displayed without applying the primary antibody in immunohistochemistry. **A–D** Bar, 50 μm. **E** and **F** Bar, 100 μm.

**Figure 4:**
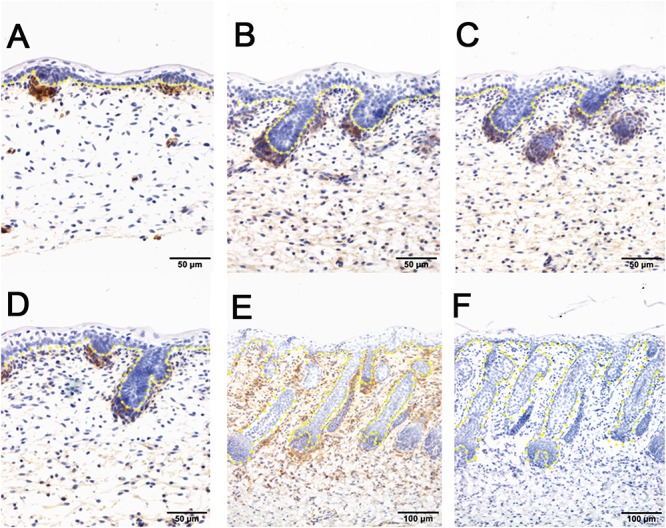
The localization of PDGFRB by immunohistochemistry is positive in dermal-originated cells and negative in apocrine sweat glands in the prenatal sheepskin. **(A)** PDGFRB is highly expressed in the dermal condensates of primary wool follicles and occasionally in dermal fibroblast before apocrine sweat gland induction; **(B–D)** PDGFRB is expressed in dermal condensates of primary and secondary wool follicles, and not in the precursor cells or the bud of apocrine sweat glands; **(E)** Strong PDGFRB expression is detected in the upper dermis of the sheepskin, especially the location surrounding the wool follicles. PDGFRB is surprisingly negative in the dermal papilla of the well-developed wool follicles and also negative in the apocrine sweat glands. **(F)** The negative control is displayed without applying the primary antibody in immunohistochemistry. **A–D** Bar, 50 μm. **E** and **F** Bar, 100 μm.

To further explore the enriched candidate genes in our data that were potentially involved in apocrine sweat gland morphogenesis, four antibodies against SOX2 (hair follicle dermal papilla marker), SOX9 (hair follicle stem cell marker), PDGFRB (platelet derived growth factor receptor beta, skin dermal development related) and SMAD5 (BMP signaling) were used to localize the protein expressions during the development of apocrine sweat glands by immunohistochemistry. SOX2 was detected specifically in the dermal condensates (DC) of primary wool follicles in early stages and in the dermal papilla (DP) of well-developed primary wool follicles in later stages (Figure 3). The apocrine sweat gland was negative for SOX2 staining (Figure 3). It is interesting that SOX2 was only expressed in the DC or DP of primary wool follicles, but not in the secondary wool follicles (Supplementary Figure S1). PDGFRB, a cell surface tyrosine kinase receptor, was observed with strong expression in dermal condensates in primary and secondary wool follicles in early stages, but with weak expression in those of well-developed wool follicles (Figure 4). The positive staining was also detected in the dermis across the whole development, with weak expression in early stages and strong expression in the upper dermis, especially the area surrounding the wool follicles and gland ducts after the dermal papilla started to form (Figure 4D,E). The apocrine sweat glands have been shown negative for PDGFRB staining during all the stages detected. These results suggested that SOX2 and PDGFRB had no direct effect on the induction of sweat gland buds in sheepskin.

**Figure 5:**
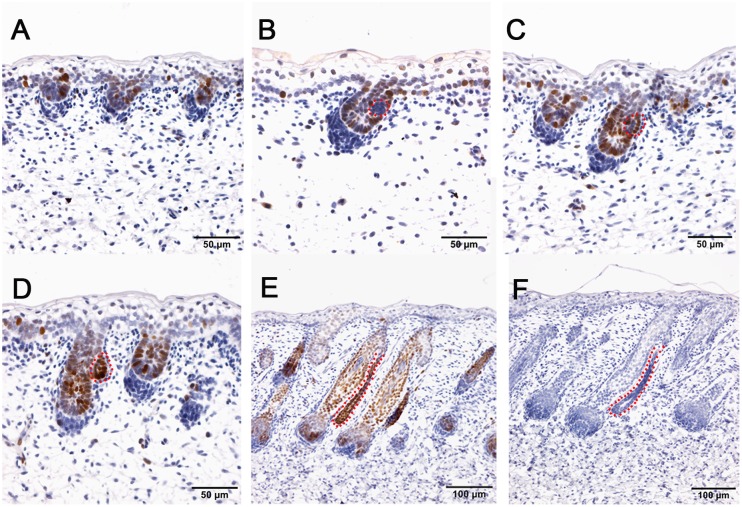
SOX9 is localized in the wool follicle pegs and apocrine sweat glands (red dashed line). **(A)** SOX9 is expressed in the placodes of primary wool follicles and occasionally in the basal layer; **(B)** As the primary wool follicles grow downward into the dermis, SOX9 is strongly expressed in the primary wool follicle pegs and basal layer, and weakly in the precursor cells of the apocrine sweat glands; **(C–D)** SOX9 is expressed in the apocrine sweat gland budding point **(C)** and buds **(D)**, and also in wool follicle pegs. **(E)** SOX9 is strongly expressed in the ducts of apocrine sweat glands. It is also expressed in the elongated epidermal compartments of wool follicles. **(F)** The negative control is displayed without applying the primary antibody in immunohistochemistry. **A–D** Bar, 50 μm. **E** and **F** Bar, 100 μm.

The hair follicle bulge stem cell marker *SOX9*, was reported to express in a population of outer root sheath cells (Nowak et al., 2008; Rompolas and Greco, 2014; Purba et al., 2015). In sheepskin, SOX9 was detected with occasional staining in the basal layer of the epidermis and strong signals in the highly proliferated and differentiated epidermal compartments of wool follicles during the early and later stages (Figure 5). Strong SOX9 signals were also detected in the apocrine sweat glands, from the precursor cell patches, to the budded and elongated apocrine sweat gland ducts (Figure 5B–E). More details showed that SOX9 was first detected with weak expression in the cell aggregates that indicated the precursor/progenitor cells of apocrine sweat glands (Figure 5B). Strong expression of SOX9 was then observed initially in budding sites, later in the germs and the elongated ducts of the apocrine sweat glands (Figure 5C–E).

**Figure 6:**
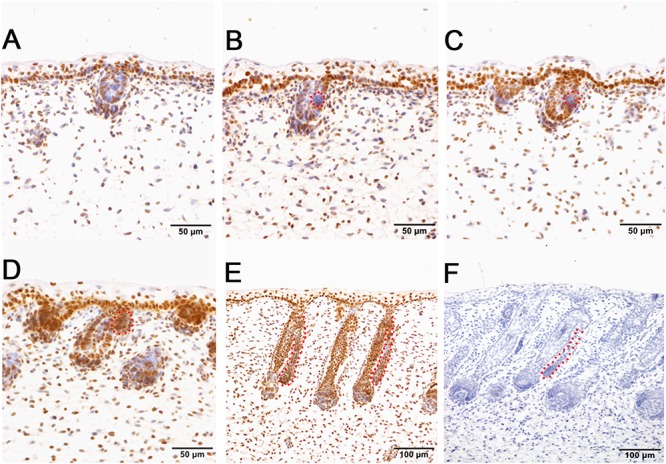
pSMAD5 is broadly expressed in the compartments of prenatal sheepskin. **(A)** pSMAD5 is expressed in the dermis, epidermis and primary wool follicle placodes and associated dermal condensates. **(B–E)** pSMAD5 is expressed in the precursor cells, buds and ducts of apocrine sweat glands and is also expressed in all skin compartments. **(F)** The negative control is displayed without applying the primary antibody in immunohistochemistry. **A–D** Bar, 50 μm. **E** and **F** Bar, 100 μm.

Immunohistochemistry of pSMAD5 showed broad expression in sheepskin across the developmental stages. The epidermis and dermis in addition to the wool follicles and sweat glands were all positive for pSMAD5 antibody staining (Figure 6). Detailed inspection revealed that the strongest positive signals of pSMAD5 were observed in the basal layer of the epidermis and the epidermal compartments of wool follicles during all the stages detected. During the apocrine sweat gland development, pSMAD5 antibody was localized initially in the few pre-gland precursor/progenitor cells with weak expression (Figure 6B,C), and then in the budded gland loops and later in the elongated gland ductal portions with strong expression (Figure 6D,E).

## Discussion

**Figure 7:**
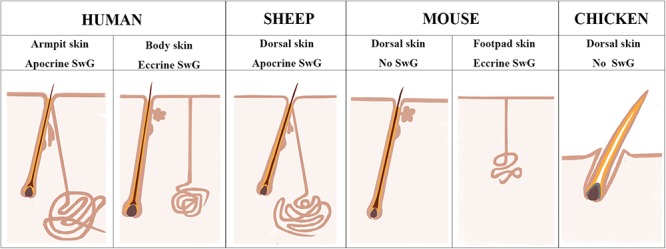
Sheepskin is an additional system to study human apocrine sweat glands. A diagram is drawn up to compare the complexity of sweat glands in human skin with those in the widely used animal models (mouse and chicken) and also in sheep used in current study. There are two types of sweat glands that exist in human skin. Eccrine sweat glands are distributed in hairless skin regions, while apocrine sweat glands are exhibited in the armpit skin. Sheep dorsal skin contains apocrine sweat glands that are similar with those of human armpit skin. Mouse dorsal skin has no sweat glands. Eccrine sweat glands exist exclusively in mouse footpad skin. Chicken skin is completely absent of sweat glands.

Chicken and mouse are the most widely used models to study the mechanisms underlying skin and feather/hair follicle morphogenesis, development, cycling and regeneration. Though lots of genetically modified mouse/chicken models were created to perform the functional study of individual or combined genes or gene networks in skin and feather/hair research, there are still lots of questions that remain unclear due to the limitation of animal models themselves. Chicken skin consists of only feather follicles with no sweat glands or sebaceous glands (Figure 7). Hair follicles and sebaceous glands are the two appendages that exist broadly in mouse dorsal skin, while eccrine sweat glands as another appendage remain specifically in mouse paw skin (Figure 7). This additional and regional localization of eccrine sweat glands in footpad skin is a good point to study the subtype appendage determination during the early development of skin. The hair follicles in mouse dorsal skin display three synchronized developmental waves prenatally and cyclic growth pattern postnatally. Moreover, numerous genetic modified mouse models were generated in recent years, benefiting from the profoundly developed genome editing technique. All the advantages contribute to making the mouse model a broadly used system for deep understanding of the skin biology.

Human skin is distinctive from other animals partially in that the sweat glands exist across the skin and the subtypes of sweat glands are either eccrine sweat glands on the non-hairy area or apocrine sweat glands on the hairy area (Figure 7). Hence the absence of apocrine sweat glands in mouse and chicken skin restrains the further understanding of the complexity of human skin biology and skin diseases, like armpit and body odor. Sheep could serve as an additional system to further explore the knowledge of apocrine sweat glands since sheepskin has sweat glands that are similar to those located in human armpits (Figure 7). Until now, there has been good understanding of the development of eccrine sweat glands (Klaka et al., 2017; Kurata et al., 2017), but not that of apocrine sweat glands. Our study is the first report revealing the complex molecular network regulating early development, especially the morphogenesis of apocrine sweat glands using sheep as a model. Coarse wool sheep develop primary and secondary wool follicles that are similar to the generation of hair follicles in mouse dorsal skin. The occurrence of apocrine sweat glands is potentially in accompaniment with the generation of secondary wool follicles as indicated in Figure 1C. The observation that the eccrine sweat glands were initiated to form the first wave of pre-germ at E16.5 (the period of secondary follicles emergence) in the proximal footpad and later at E17.5 in the distal footpad (Schlake, 2007; Cui et al., 2014) implied that the two types of sweat glands shared the similar induction time schedule during the early morphogenesis.

At the induction stage, a few cells packing together on the lateral side of the half length of primary wool follicle germ indicated the location of precursor/progenitor cells of apocrine sweat glands approximately at embryonic day 75 (Figure 1C). This unilateral pattern formation is different from the bilateral pattern of sebaceous glands that develop after the apocrine glands in sheepskin. It is also different from the *de novo* pattern formation of eccrine sweat glands that develop from the crosstalk of epidermal and dermal layers of the skin (Lu et al., 2016). These compact cell patches gradually grew outward from the adjacent outer root sheaths of primary wool follicles to form the short and later long ductal bud as indicated by the H&E stain in our study (Figure 1). Then the bud extended closely parallel to the primary wool follicle peg and developed the secretory portions to become mature apocrine sweat glands in later stages.

The two developmental stages applied in the RNA-sequencing program represent the pre-gland phase (stage TF1b, Figure 1B) and gland bud phase (stage TF2a, Figure 1E) of apocrine sweat glands. At the selected gland budding stage, the primary wool follicles grew downward to the dermis to develop follicle pegs and at the same time secondary wool follicles started to generate the placode and associated dermal condensates. Briefly, the prominent structural changes between the two stages are the thickened epidermis and dermis, elongated wool follicle pegs, enlarged dermal condensates, initiated secondary wool follicle placodes and emerged apocrine sweat gland germs. By analyses of 1631 DEGs, a series of genes was enriched to function in skin development (33 genes) and hair follicle development (14 genes) (Table 2). These genes are potentially responsible for the morphological changes for skin epidermal and dermal thickening as well as wool follicle germ elongation. Most of the genes showed the increased expression trends in line with the positive regulation of cellular processes and biological processes in enriched GO terms. Further analyses showed that our data were highly comparable with those of genes regulating the hair follicle development in P5 mouse skin. The overlapped genes between these two groups covered regulatory genes responsible for all the compartments of the skin and hair/wool follicles. Genes regulating each compartment of wool/hair follicles were analyzed and recorded in Table 1 and represented by *TGFB1* and *WNT16* for epidermal development, *ADAMTS15* and *COL6A1* for the dermal fibroblast development, *LAMA5* and *SOX9* for outer root sheath development, *LRIG1* and *SOX9* for hair follicle stem cell development. These results indicate that the epidermal part of wool follicles was rapidly growing during the two stages we detected. The most promising result is the enrichment of 34 genes for wool follicle dermal papilla development represented by the commonly used dermal condensate marker genes *BMP3*, *SOSTDC1*, *TRPS1* and *WIF1*. These analyses were consistent with the enlarged dermal condensates associated with the primary follicle pegs and the newly formed secondary wool follicles as shown in Figure 1C.

Though these two datasets do not originate from the same developmental stages, they do share similar gene networks that govern the skin and hair/wool follicle development between mouse and sheep. It indicates that the pre-mature wool follicles in the selected stages of sheepskin and mature hair follicles in P5 mouse skin were regulated by partially conserved candidate genes with different dosages or locations. The overlapping marker genes in Table 1 and the immunohistochemistry of selected candidates in Figure 3–6 clearly stated this notion.

SOX2, a hair follicle maker, was specifically positive in the dermal condensates/dermal papilla of the primary wool follicles and surprisingly negative in the secondary wool follicles (Figure 3 and Supplementary Figure S1). This expression pattern is partially different from those of the mice in that Sox2 was detected in both primary and secondary hair follicles, not in the third wave zigzag follicles in mouse dorsal skin (Graham et al., 2003; Driskell et al., 2009). PDGFRB is one of the types of PDGF receptors which can mediate the biological actions of PDGF and is related to the development of many organs (Claesson-Welsh et al., 1988; Gronwald et al., 1988; Mellgren et al., 2008). Moreover, Pdgfrb was expressed in the dermis and dermal condensates of E14.5 mouse skin (Rezza et al., 2015) and disruption of *Pdgfrb* signaling impaired proliferation and dermal fibroblast migration (Gao et al., 2005; Rajkumar et al., 2006). In sheepskin, PDGFRB displayed focal expression in the dermal papilla of the pre-mature wool follicles and the fibroblast in early stages, and later in the upper part of the mesenchyme, especially the area surrounding the wool follicles. The signals of SOX2 and PDGFRB were both absent from the ductal buds of the apocrine sweat glands, indicating these two genes were not important for the early morphogenesis of apocrine sweat glands. And it also suggests that the regulatory networks of dermal-originated hair/wool follicle compartments were different from those of apocrine sweat glands branched from the epidermal-originated outer root sheaths of the wool follicles. Sox9 was reported to mainly express in the outer root sheath and the bulge of hair follicles (Nowak et al., 2008; Rompolas and Greco, 2014; Purba et al., 2015). The detection of SOX9-positive signals in epidermal compartments of the wool follicles, the inter-follicular basal layers and the apocrine sweat gland ducts implied that hair/wool follicles and apocrine sweat glands partially share some key regulators, especially the molecules regulating the outer root sheaths during the morphogenesis. The expression of SOX9 was initially detected in the precursor cells (the cell aggregates located on the lateral side of the wool follicle peg) of the apocrine sweat gland and later in the branched gland germs and straight gland ducts (Figure 5). The aggregate precursor cells marked the initiation of apocrine sweat glands. Then these few cells proliferate, differentiate and migrate to the edge of the follicle peg and form the gland cavity with the small opening to the upper part of the outer root sheath.

The induction of apocrine sweat gland germs and the elongated epidermal compartment of wool follicle pegs are highly correlated with the epithelial cell migration, differentiation, and morphogenesis of epithelial branching or tube formation (Table 2). The enrichment of 7 categories of genes involved in epithelial branching or tube formation is consistent with the observation that the wool follicle is tube-like in structure, branching from the skin basal layer, and the apocrine sweat glands protrude from the outer root sheath to form the branching with two layers (basal layer and supra-basal layer) surrounding the cavity of the gland (Figure 1I). These structures mostly originate from the epithelia in line with the enrichment of epithelial-related GO terms and candidate genes.

The most interesting point is the enrichment of 5 gland-related GO terms in our data. A series of genes represented by *BMP7*, *FGFR1*, *GLI2*, *LAMA5*, *SOX9*, and *WNT5A* enriched in gland morphogenesis (18 genes increased and 2 genes decreased) and gland development (34 genes increased and 8 genes decreased) were reported to be involved in general gland development, including the salivary gland, mammary gland and prostate gland development. Though the apocrine sweat gland in our study is structurally different from those types of glands mentioned above, they do partially share the regulatory genes functioned in the early morphogenesis, especially the ductal formation. A total of 8 genes (*BMP7*, *DAG1*, *EGFR*, *FGFR1*, *LAMA5*, *NRP1*, *SNAI2*, and *TGFB3*) were grouped specifically for salivary gland morphogenesis and development, while 6 genes (*BMP7*, *DAG1*, *FGFR1*, *LAMA5*, *NRP1*, and *SNAI2*) were enriched for branching involved in salivary gland morphogenesis. Recently, a report suggested that conditional deletion of *Nrp1* in mammary epithelial cells delayed mammary development, particularly the ductal extension (Liu et al., 2017). Lama5 and Dag1 were broadly expressed in the basement membrane of skin and hair follicles in mouse models to maintain skin integrity. Previous reports showed that *Lama5* played important roles in hair peg elongation and skin homeostasis since conditional knockout mice *Lama5^Ker5^* showed delayed hair growth in early age, abnormal follicle down-growth and decreased hair follicle density in adult animals (Wegner et al., 2016). The fact that LAMA5 was highly expressed in the basement membrane of straight ducts and secretory portions of the human eccrine sweat glands (Kurata et al., 2017) implied that the increased expression of *LAMA5* in the apocrine sweat gland budding stage was responsible for both the wool follicle peg elongation and the ductal formation of the apocrine sweat glands. *SNAI2* (*Slug*) was shown to determine the mammary stem cells in line with Sox9 (Guo et al., 2012). The upregulation of *SNAI2* in our data indicates that *SNAI2* was involved in apocrine sweat gland induction. These analyses further proved the notion that the molecular networks controlling gland morphogenesis were partially shared among diverse glands (salivary gland, mammary gland and apocrine sweat gland) and functionally different from those of hair/wool follicles development.

In addition to the GO terms mentioned above, we also significantly enriched three signaling pathways (WNT, TGF-β, and Hedgehog) that may be involved in apocrine sweat gland morphogenesis and development. In our data, we accumulated 19 genes of the WNT signaling pathway. The WNT pathway was indispensable for induction and development of hair follicles and eccrine sweat glands (Chen et al., 2012; Cui et al., 2014). Among these genes, *Wnt5a* was involved in the proper development of bud outgrowth and branching point formation of the prostatic gland (Huang et al., 2009b). The application of Wnt5a protein in a tissue culture system also inhibited the ductal branching and extension of mammary gland (Roarty and Serra, 2007). During this process, *Wnt5a* was supposed to function as a downstream effector of TGF-β signaling that showed similar regulatory impact on mammary gland development (Roarty and Serra, 2007). The upregulation of *WNT5A* in our data indicated that *WNT5A* is involved in early development of apocrine sweat gland. But whether or not *WNT5A* inhibits the formation of apocrine sweat gland duct requires further study. *BMP7*, *BMPRIA*, *SMAD1*, and *SMAD4* were enriched in the TGF-β signaling pathway. *Smad1* was focally expressed in the dermis of eccrine sweat gland germs during the induction stage (O’Shaughnessy et al., 2004). *Smad4* is a common partner interacting with *Smad1*, *Smad5*, and *Smad8* to mediate BMP signaling, and with *Smad2* and *Smad3* to mediate TGF-β subfamily signaling (ten Dijke and Hill, 2004). *Smad4* conditional knockout mice exhibited abnormal proliferation and differentiation, particularly with increased cell proliferation in the outer root sheaths and epidermis, mainly due to the blockage of TGF-β subfamily (Owens et al., 2008). Several mouse models reported that upregulation of BMP signals was important for eccrine sweat gland development. The overexpression of *Noggin* (antagonist of BMP) in *K14-Noggin* transgenic mice displayed increased hair follicle density in body skin, and transformed eccrine sweat glands in footpads into hair follicles (Plikus et al., 2004). The *Bmpr1a* conditional knockout mice converted the eccrine glandular appendage fate to form hair follicle-like structures in mouse footpad skin (Lu et al., 2016). These mouse models related to the BMP pathway highly suggest that upregulation of BMP signals favor eccrine sweat gland development. *Bmp7*, a conserved secreted molecule of BMP family, was detected in both epidermal and dermal layers in mouse E13.5 skin, broadly expressed in the epithelium of the salivary gland, the immature hair follicles (inner and outer root sheaths, hair shaft and dermal papilla) and the mesenchyme surrounding the hair follicles, and highly enriched in mature hair follicles (dermal papilla and outer root sheaths) (Zouvelou et al., 2009). The *Bmp7* conditional knockout mice showed abnormal hair follicles with enlarged root sheaths. *BMP7* also proved to regulate the branching of lacrimal glands and prostate glands (Dean et al., 2004). The increased expression of *BMP7* in our data, combined with the reported functional study of *BMP7* in gland development and the expression pattern of *BMP7* in the outer root sheath of hair follicles, strongly suggest that *BMP7* was potentially involved in the morphogenesis of apocrine sweat glands. In our study, the localization of pSmad5 was observed at the branching point and the germs of the apocrine sweat glands with strong expression (Figure 6). This expression pattern of pSmad5 suggested that BMP signaling was activated at the gland induction site as that of the eccrine sweat gland. Hence, the enrichment of TGF-β, particularly BMP-signaling genes *BMP7*, *BMPRIA*, *SMAD1*, and *SMAD4* in our sequencing analysis, combined with the mouse models of the BMP pathway discussed above, highly suggest that BMP signaling is a positive regulator of apocrine sweat gland induction.

The competence of high-BMP and low-SHH signals in a short developmental period established the initiation of eccrine sweat glands instead of the hair follicles in mouse ventral foot pad skin as discussed previously (Lu et al., 2016). Interestingly, the SHH signaling pathway was enriched in our data as well, including genes *SHH*, *GLI2*, and *GLI3*. The SHH signaling pathway was shown to positively regulate the down-growth of hair follicle pegs and negatively determine the induction of eccrine sweat glands in a short developmental stage (Cui et al., 2011, 2014; Lu et al., 2016). The increased expression of SHH signaling in our data was potentially responsible for wool follicle elongation and gland duct extension. It is also possible that SHH regulated the induction of apocrine sweat glands at the earlier stage and at the restricted branching point.

The histological study in our current report clearly stated that apocrine sweat glands in sheepskin were branched from the outer root sheaths of the primary wool follicles. A total of 43 genes enriched in 5 categories of gland morphogenesis and development in our data, implying that the regulatory network for the morphogenesis of apocrine sweat glands in sheepskin was partially conserved with the other glands, particularly mammary glands, salivary glands and eccrine sweat glands, though the originations of apocrine sweat glands and eccrine sweat glands are different. Of those, the BMP and WNT signaling pathway genes (*BMP7*, *BMPR1A*, *SMAD1*, *SMAD4*, and *WNT5A*) and the 8 gland-related genes are the most promising candidates potentially exhibiting positive regulation of apocrine sweat gland induction. The negative regulators during this process are not specified in our data. It may be that SHH pathway genes (*SHH*, *GLI1*, and *GLI2*) functioned in the branching point during the induction of apocrine sweat glands. Until now, few studies have been conducted on apocrine sweat gland development. Moreover, transcriptome studies of sheepskin have also been widely reported, but little attention has been paid to the development of apocrine sweat glands. Our report is the first to reveal the complex molecular network interaction in the induction stage of apocrine sweat glands in coarse wool sheepskin and will contribute to the better understanding of the histology, physiology and pathology of apocrine sweat glands and associated diseases in humans and companion animals.

## Data Availability Statement

The RNA-seq data were submitted to the NCBI database under the SRA Accession: PRJNA507468.

## Author Contributions

CM designed the experiment, wrote and revised the manuscript. SL wrote the manuscript, analyzed the data, and performed the qRT-PCR and immunohistochemistry. XZ, YN, and MY involved in sample collection and staining. WC, YT, and XH adjusted the picture format. ZL, YH, HQ, QQ, QP, and DC participated in the collection of samples.

## Conflict of Interest Statement

The authors declare that the research was conducted in the absence of any commercial or financial relationships that could be construed as a potential conflict of interest.
