# Supplementary material for: Defining Key Genes Regulating Morphogenesis of Apocrine Sweat Gland in Sheepskin
Source: Front Genet. 2019 Jan 30;9:739. doi: 10.3389/fgene.2018.00739 (PMC6363705; doi:10.3389/fgene.2018.00739)
Supplement: Supplementary file 4 [file Data_Sheet_1.pdf]

Supplementary Material

Defining Key Genes Regulating Morphogenesis of Apocrine Sweat Glands in Sheepskin

Shaomei Li[1], Xinting Zheng[1], Yangfan Nie[1], Wenshuo Chen[1], Zhiwei Liu[1], Yingfeng Tao[1], Xuewen Hu[1], Yong Hu[2], Haisheng Qiao[2], Quanqing Qi[3], Quanbang Pei[3], Danzhuoma Cai[4], Mei Yu[1] and Chunyan Mou[1]*

***Correspondence:**

Chunyan Mou

chunyanmou@mail.hzau.edu.cn

1 Supplementary Figures

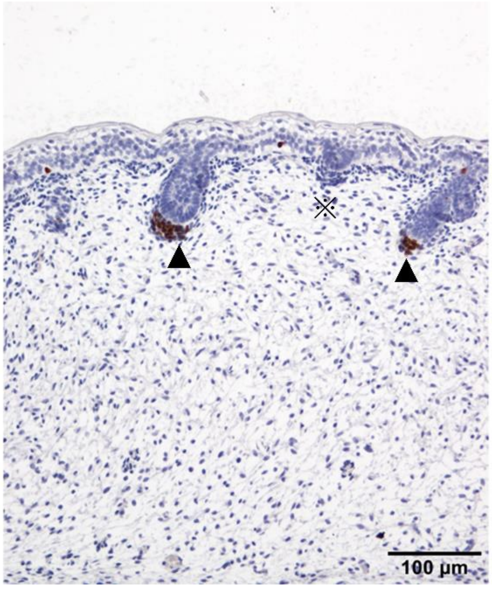

Supplementary Figure 1 SOX2 is exclusively expressed in dermal condensates of primary wool follicles, not in secondary wool follicles.▲, primary wool follicle; ※, secondary wool follicle.

Supplementary Table 1 Differentially expressed transcripts between stage TF2a and stage TF1b of apocrine sweat gland induction in carpet wool sheep fetal skin.

Supplementary Table 2 Significantly enriched GO terms of differentially expressed genes.

Supplementary Table 3 Significantly enriched KEGG pathways of differentially expressed genes.
